# Treatment for dental erosion: a systematic review of *in vitro* studies

**DOI:** 10.7717/peerj.13864

**Published:** 2022-11-08

**Authors:** Yago Gecy de Sousa Né, Deiweson Souza-Monteiro, Deborah Ribeiro Frazão, María Olimpia Paz Alvarenga, Walessa Alana Bragança Aragão, NatháliaCarolina Fernandes Fagundes, Renata Duarte de Souza-Rodrigues, Rafael Rodrigues Lima

**Affiliations:** 1 Laboratory of Functional and Structural Biology, Institute of Biological Sciences, Federal University of Pará, Belém, Pará, Brazil; 2 Faculty of Medicine and Dentistry, University of Alberta, Edmonton, Canada

**Keywords:** Tooth erosion, Treatment, Systematic review

## Abstract

**Background:**

Dental erosion is a chemical loss of the mineralized dental tissue caused by exposure to nonbacterial acids. Different treatment protocols have been adopted with the use of fluoride compounds to promote the formation of a layer of mineral precipitation in eroded lesions.

**Aim:**

This systematic review aimed to evaluate the main treatments for dental erosion.

**Methodology:**

This study followed the Preferred Reporting Items for Systematic Reviews and Meta-Analyses (PRISMA) guidelines and recorded in the Open Science Framework database (OSF) under DOI 10.17605/OSF.IO/XMFNZ. The searches were conducted in six electronic databases (Pubmed, Embase, Web of Science, Cochrane, Scopus, Lilacs) and two grey literature sources (Google Scholar and OpenGrey). The eligibility criteria included *in vitro* studies that evaluated eroded teeth under treatment with some topical agent. Risk of bias assessment and qualitative synthesis were performed using the Cochrane collaboration’s tool for assessing risk of bias modified for *in vitro* studies.

**Results:**

A total of 522 studies were identified, and only four studies that fulfilled our eligibility criteria were included in this review. Among these studies, three were considered to have a low risk of bias, and one to have a high risk of bias. Two studies evaluated the anti-erosion effect of fluoride toothpaste, and the other two assessed the action of casein phosphopeptide–amorphous calcium phosphate (CPP-ACP) on the surface of human teeth. Among the products analyzed, CPP-ACP was the only one that promoted a significant increase in enamel microhardness and reduced tooth wear.

**Conclusion:**

Based on the *in vitro* studies included in this review, there was no anti-erosion effect after using different fluoride toothpaste. However, it should be considered that one of these studies presented a high risk of bias. On the other hand, studies with CPP-ACP showed anti-erosion efficacy when applied before or after erosive wear.

## Introduction

Dental erosion is defined as the chemical loss of dental mineralized tissue caused by exposure to nonbacterial acids (Schlueter et al., 2020). This is a multifactorial condition whose etiology and pathogenesis are related to chemical, biological, and behavioral factors (Kanzow et al., 2016). These factors, acting individually or interacting with each other, play a role in the prevention or progression control of dental erosion (Lussi, 2006).

The early diagnosis of erosive lesions is still challenging for most professionals and is usually identified as the rapid progress of tooth wear where there is already dentin hypersensitivity and the absence of lesion staining (Donovan et al., 2021). Also, other cases of erosive wear occur in the process of slow progression, which does not generate symptoms due to the response mechanism of the dentin-pulp complex that promotes the obliteration of dentinal tubules (Bartlett, 2016).

In this context, direct exposure to acids in the oral cavity promotes the demineralization of hydroxyapatite due to the undersaturation of minerals concerning the surrounding microenvironment (Shellis, Featherstone & Lussi, 2014). Therefore, saliva plays an essential role in the bioavailability of calcium and phosphate ions for the remineralization process. However, the frequent action of acids can overcome the protective effect of saliva, causing mineral dissolution that results in erosive wear (Fernando et al., 2019). To reverse this process, it is necessary to use topical remineralizing agents that contain calcium, phosphate, and mainly fluoride ions (Shen et al., 2018).

The use of fluoride in the form of mouthwash or brushing with toothpaste promotes structural remineralization, forming a mineral layer on the tooth surface and reducing subsequent demineralization, thus helping in the treatment and prevention of dental erosion (Creeth et al., 2015). Although fluoride products are often used, most fluoridated formulations alone have limited preventive effects against tooth erosion as their action on the mineralization process is limited to the surface and the near-surface layers of enamel and is restricted to the demineralized enamel layer (Lussi & Carvalho, 2015).

Numerous vehicles, such as toothpaste, rinse solutions, gels, and varnishes, are currently available as strategies for using fluorides. These products contain different types of active ingredients with distinct anti-erosive properties, and some of them have been demonstrated to be effective preventive therapies against tooth erosion (Buzalaf, Magalhães & Wiegand, 2014; Lussi et al., 2019).

The most appropriate way to access the protective effects of these products would be *in vivo* studies, with randomized clinical trials considered the gold standard. However, this kind of study has some limitations, such as the low accuracy of available methods for measurement of chemical tooth tissue loss, the need for prolonged studies to assess the rate of progression and the variation of this rate, the lack of control over the exposure to wear, the need for participation of large groups, to minimize contact with other erosive agents, and for several years of follow-up (West, Davies & Amaechi, 2011). Proper clinical methods must be developed and validated to assess dental erosion variables *in vivo*. Numerous *in vitro* model studies have been designed to simulate everyday intra-oral conditions as closely as possible (Wiegand & Attin, 2011). Thus, this systematic review was carried out to gather scientific evidence demonstrating the anti-erosion treatments that presented the best results in the remineralization of the tooth structure.

## Materials & Methods

### Registration

This study was recorded in the Open Science Framework database (OSF) under registration DOI 10.17605/OSF.IO/XMFNZ and performed according to the guidelines of the Preferential Reporting Requirements for Systematic Review (PRISMA) (Page et al., 2021).

### Eligibility criteria

This systematic review was guided by the PICO (P = patient or participant, I = intervention, C = comparison, O = outcome) question strategy, covering studies in eroded teeth (P) treated with any topical agent (I) in comparison without treatment (C), whose primary outcome was the change in enamel tissue (O).

The eligibility criteria included studies with human teeth and permanent teeth, in which *in vitro* data was recorded after tooth erosion and used any topical agent with an active ingredient that could be used for the treatment of dental erosion and compared with the group that used artificial saliva. Studies that did not fall within the PICO were excluded as well as technical papers, clinical cases, literature reviews, uncontrolled studies, guides, letters to the editor and opinion articles. Also, studies that evaluated bovine teeth were excluded because they had different histological characteristics from human teeth. Studies not using specimens placed in artificial saliva as a comparison group were also excluded.

### Search and selection of studies

A search strategy composed of keywords and free terms was used to search for studies in the electronic databases Pubmed, Web of Science, Lilacs, Scopus, Embase and Cochrane, as well as the grey literature search through Google Scholar and OpenGrey (Table S1). After that, a reference manager (EndNote, version X9, Thomson Reuters, Philadelphia, USA) was used to exclude duplicate citations.

The first stage of the study selection was carried out by reading the titles and abstracts. The remaining articles were read in full to verify eligibility. From this stage, the papers selected for this review were defined, and a manual search was carried out in the references in order to find other eligible articles. Two independent authors performed the search and selection of studies, and a third reviewer evaluated the data in cases of disagreement.

### Data extraction and risk of bias analysis

The following data were extracted from each study: authors, country of publication, study type, tooth type, erosion assessment, erosion treatment, and results. In cases where some data were missing, the authors were contacted by e-mail once a week for a month until the information was obtained.

After the data extraction, two independent researchers performed the risk of bias assessment. At the same time, the disagreements were resolved by a third researcher in consensus with the peers. The Cochrane collaboration’s tool (Higgins et al., 2011) was applied to assess the risk of bias. The selected studies were processed in the Review Manager software (version 5.3, Copenhagen: The Cochrane Collaboration, https://training.cochrane.org/online-learning/core-software/revman).

Randomized sequence creation, allocation concealment, participant blinding, blinding of outcome assessment, inadequate outcome data, and selective reporting are the six major bias domains covered by this instrument. For the final evaluation of each study, the following essential domains were chosen: concealment of selection, blinding of participants, and blinding of outcome evaluation. A study with a high risk of bias was defined as having severe issues in two key domains (Table S2).

## Results

### Characteristics of the studies

A total of 522 records were identified from the database search, and 305 duplicates were found and removed. The remaining 217 were evaluated by title and abstract according to the eligibility criteria, and as a result, 204 studies were excluded at this stage.

**Figure 1:**
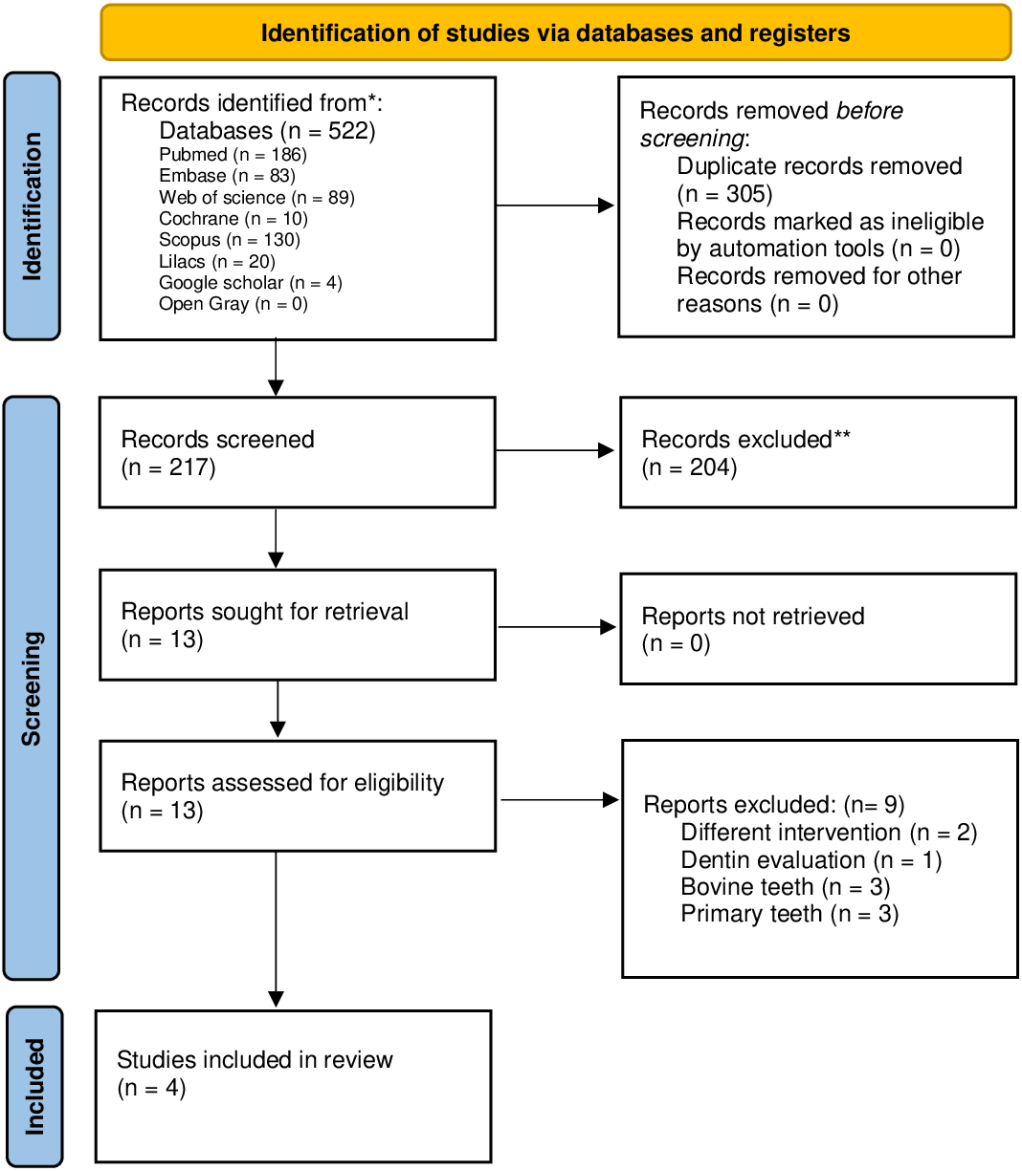
Flowchart of the study selection process according to the PRISMA protocol. The asterisks (*, **) indicate the number of points attributed to each domain in the quality assessment to each article.

The remaining studies (*n* = 13) were assessed by full-text reading: three used bovine teeth (Comar et al., 2015; Machado et al., 2019; Scandiffio et al., 2018), and one evaluated dentine (João Souza et al., 2020). Two studies did not realize the intervention group (Austin et al., 2014; Passos, Rodrigues & Santiago, 2018), and three evaluated primary teeth (Elwardani, Harhash & Zaky, 2019; Maden et al., 2017; Rallan et al., 2013), conflicting with the previously established eligibility criteria. Finally, four studies were selected in this systematic review according to the eligibility criteria (Bradna et al., 2015; João Souza et al., 2017; Panich & Poolthong, 2009; Ranjitkar et al., 2009) (Fig. 1).

The four included studies were *in vitro* studies. One was conducted in the Czech Republic (Bradna et al., 2015), one in Brazil (João Souza et al., 2017), one in Thailand (Panich & Poolthong, 2009), and one in the United Kingdom (Ranjitkar et al., 2009). These studies evaluated the surface of eroded tooth enamel and analyzed the effect of different remineralizing treatments.

### Individual results of studies

The substances used to induce erosive wear were citric acid solution (Bradna et al., 2015; João Souza et al., 2017; Ranjitkar et al., 2009) and Coca-Cola (Coke®) drink (Panich & Poolthong, 2009). The studies by Bradna et al. (2015) and João Souza et al. (2017) evaluated the effect of various commercial toothpaste compared to the control group that immersed the teeth only in artificial saliva. In the first study mentioned, the treatment groups used fluoride-based (Sensodyne® Pronamel), toothpaste with stannous fluoride (Elmex® Erosion Protection), and toothpaste with calcium (BioRepair plus sensitivity control, SensiShield, Enamel Care). The second study evaluated several desensitizing and/or anti-erosion toothpaste (Table 1). These two studies’ results revealed that toothpaste’s anti-erosion effectiveness is associated with the presence of tin, high concentrations of phosphate and calcium, strong potential to form acid-resistant deposits on the enamel surface, and low potential to create acid-resistant deposits on the enamel surface abrasivity.

Two studies included in this systematic review showed the use of fluorinated or non-fluorinated tooth mousses containing casein phosphopeptide-amorphous calcium phosphate (CPP-ACP) (Panich & Poolthong, 2009; Ranjitkar et al., 2009), which is a bioactive agent derived from milk protein that can stabilize calcium phosphate in the tooth structure (Gokkaya et al., 2020). Thus, considering the variability of anti-erosion treatment protocols between the two studies, it can be identified that oral products containing CPP-ACP presented the best results in reducing erosive enamel loss expressed by increasing surface microhardness and reducing surface roughness.

The studies, in general, showed that pastes with a higher concentration of ions such as calcium and phosphate and smaller particle size showed greater effectiveness in protecting against dental erosion so that acids could not degrade the mineral matrix. Among the topical agents, acidulated phosphate fluoride (APF) gel, Elmex® erosion, and Sensodyne® Pronamel showed the best results related to enamel protection (Bradna et al., 2015; João Souza et al., 2017; Ranjitkar et al., 2009).

**Table 1:** Data and characteristics and studies included.

Author (Year)/Country	Size and source of sample	Teeth	Erosion evaluation	Erosion treatment	Results
Bradna et al. (2015)/ Czech Republic	*N* = 35 General University Hospital in Prague, Czech Republic	Third molars	Microhardness testing	Experimental groups: Sensodyne Pronamel; Elmex erosion protection; BioRepair plus sensitivity control; SensiShield; Enamel care for sensitive teeth Control groups: Artificial saliva Elmex erosion mouth rise	Enamel Care formed a compact layer of deposits on the enamel surface, while Sensodyne Pronamel and BioRepair Plus Sensitivity Control did not produce any protective deposits. On the other hand, Elmex Erosion Protection and SensiShield showed high abrasivity and consequent low anti-erosion action.
João Souza et al. (2017)/ Brazil	*N* = 150 Department of Restorative Dentistry, University of São Paulo, School of Dentistry	Premolar	Microhardness testing	Experimental groups: Sensodyne Repair and Protect; Elmex Sensitive Professional; Sensodyne Rapid Relief; Blend-a-Med (Oral-B) Pro Expert; Sensodyne Pronamel; Elmex Erosion Protection; Candida Protect Professional; Regenerate Control groups: Artificial Saliva Colgate Caries Protection	All groups showed progressive surface loss. Sensodyne Pronamel and Elmex Erosion Protection presented the lowest values of enamel surface loss, not differing from the control groups and with no difference between them (*p* > 0.05). Regenerate and Blend-a-Med Pro Expert showed the highest surface loss values.
Panich & Poolthong (2009)/ Thailand	*N* = 40 School of Dentistry, Chulalongkorn University, Bangkok, Thailand	Central and lateral incisors	Microhardness testing	CPP-ACP (Tooth Mousse) Artificial Saliva	After remineralization, the mean microhardness increased by 13.27% in the CPP-ACP group and by 2.53% in Artificial Saliva group. Two-way ANOVA results showed no statistically significant interaction among CPP-ACP, artificial saliva and deionized water (*p* = 0.548).
Ranjitkar et al. (2009)/ United Kingdom	*N* = 36 Guy’s Hospital, London, United Kingdom	Third molars	Profilometry	Tooth Mousse (CPP-ACP) Tooth Mousse (without CPP-ACP) Artificial saliva	Tooth Mousse significantly reduced enamel wear (*p* < 0.001).

**Notes.**

APF gel Acidulated phosphate fluoride gel CPP-ACP Casein phosphopeptide-amorphous calcium phosphate CPP-ACPF Casein phosphopeptide-amorphous calcium phosphate fluoride Er,Cr:YSGG Erbium yttrium scandium gallium garnet

Nevertheless, Elmex® erosion toothpaste showed high adhesion to enamel due to the high concentration of abrasive agents such as tin, it made the enamel surface a little scratched, compared to Sensodyne® Pronamel, which showed a smoother surface indicating less abrasiveness (Bradna et al., 2015; João Souza et al., 2017).

### Risk of bias and qualitative assessment of studies

**Figure 2:**
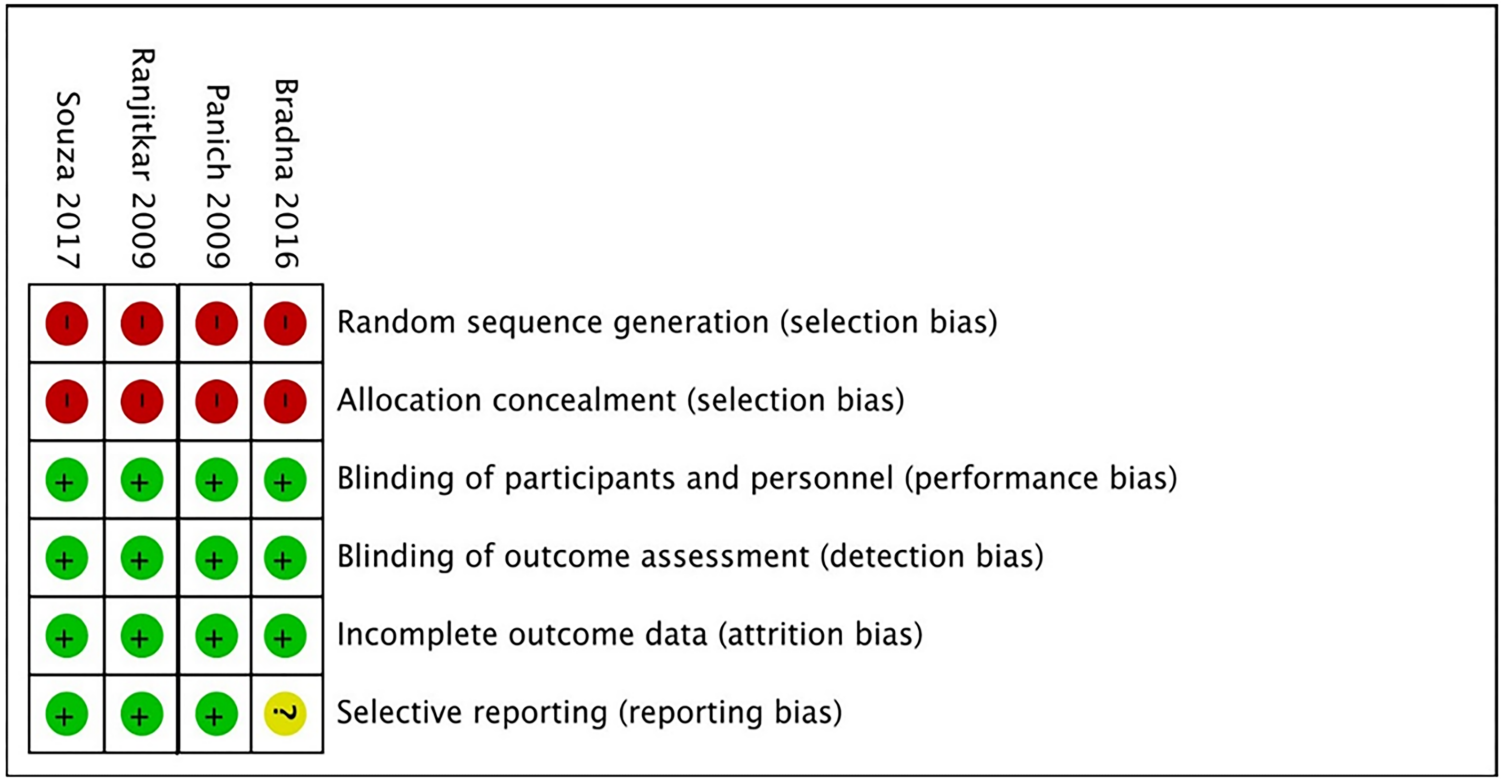
Risk of bias of the included studies, according to the Cochrane collaboration’s tool for assessing risk of bias.

In the risk of bias analysis using the Cochrane tool (Higgins et al., 2011), although all studies showed methodological problems in the domain of the test machine blinding operator, it was not significant to increase the risk of bias because the sample was large enough, so three of the four selected studies were considered to be at low risk of bias (Panich & Poolthong, 2009; Ranjitkar et al., 2009; João Souza et al., 2017) (Fig. 2). Only one of the studies demonstrated a high risk of bias due to the lack of quantitative data (Bradna et al., 2015).

## Discussion

Our systematic review aimed to investigate effective treatments for dental erosion. Although fluoride products showed no significant reduction in enamel surface loss in the studies, there was a remineralizing effect on dental enamel after using CPP-ACP. However, the number and limitations of the included studies must be considered.

Based on the present findings, it was possible to verify that all test products (toothpaste and tooth mousses) had different effects on enamel surface loss. Comparing these products can be difficult, as many intrinsic factors can influence their protective potential, the type of experimental model, and different ways of presenting the products used. The absence of a negative control group with exposure only to water can interfere with the mean difference between groups in erosion/abrasion models. However, when evaluated individually, it is possible to demonstrate that the performance of each product depends on several factors, such as the presence or absence of fluoride; the mode of action of fluoride; the mode of action of other protective agents, such as polyvalent metal ions, amino acids, peptides, proteins and polymers, and CCP-ACP (Lussi & Carvalho, 2015).

The influence of fluoride on dental erosion depends on many factors, such as higher concentration, frequency of application, and vehicle. In these cases, the efficacy of fluoride products is attributed to the formation of precipitates on the tooth surface, which acts as a protective barrier against acid impacts (Schlueter et al., 2020). Although the studies in this review did not show a significant remineralizing effect of fluoride on tooth enamel, other recent studies in the literature have shown that the application of fluoride products after acid challenge to enamel reduced enamel loss (Zanatta et al., 2020; Ionta, dos Santos & Mesquita, 2019). The studies by Bradna et al. (2015) and João Souza et al. (2017) showed no protective effect against dental erosion, although lower enamel surface loss was observed in teeth treated with fluoride dentifrices and/or rinses. These results are limited due to the action of other factors such as calcium and phosphate ion concentration, chemical characteristics of the compounds, and different application protocols.

In addition to these therapeutic approaches, the remineralizing properties of milk protein-based complexes have been investigated, which are used in the form of peptides, among which is the amorphous casein-calcium phosphopeptide complex (CPP-ACP) (Ekambaram, Said & Yiu, 2017). There is evidence that CPP-ACP provides high concentrations of calcium and phosphate ions that, when associated with fluoride, potentiate the remineralizing effect on tooth enamel (Gokkaya et al., 2020; Alexandria et al., 2020; Bejoy et al., 2020). The casein present in the complex stabilizes phosphate and calcium ions and facilitates the formation of mineral deposits on the surface of erosive lesions (Reema, Lahiri & Roy, 2014). Therefore, CPP-ACP effectively prevents erosive lesions when applied before the acid challenge by reducing the percentage of loss of surface microhardness of human enamel (Yu et al., 2018; Bejoy et al., 2020).

Although *in vitro* studies are very relevant, it should be noted that, no matter how much the methodology used in these studies tries to reproduce in greater detail what happens in the oral cavity, it will never be identical. For example, in dental erosion studies, it is impossible to reproduce the acquired pellicle, which is an essential factor in protecting the tooth surface against erosion. In addition, the studies used in the present systematic review show high heterogeneity in the methodologies used, such as substrate, stages of dental erosion, evaluation times, and erosive protocols, among others. Considering all these factors, the results found in our study need to be evaluated with caution. Moreover, we suggest future well-designed studies with better standardization of the methods, allowing comparison among them and increasing the quality of scientific evidence.

## Conclusions

Despite some methodological limitations in *in vitro* studies, including those discussed in this systematic review, the results provided imply that CPP-ACP therapies can be beneficial for dental erosion. However, these results should be evaluated with caution, and further studies are needed to establish the best treatment for dental erosion with high certainty of evidence.

##  Supplemental Information

10.7717/peerj.13864/supp-1 Supplemental Information 1 Search strategies

10.7717/peerj.13864/supp-2 Supplemental Information 2 Discretion for risk assessment of bias according to “The Cochrane Collaboration’s tool for assessing risk of bias (HIGGINS et al., 2011)

10.7717/peerj.13864/supp-3 Supplemental Information 3 PRISMA checklist

10.7717/peerj.13864/supp-4 Supplemental Information 4 The contribution that the present systematic review makes to knowledge in light of previously published articles

## Additional Information and Declarations

### Data Availability

The following information was supplied regarding data availability: This is a literature review and does not have raw data.
