# Supplementary material for: Treatment for dental erosion: a systematic review of in vitro studies
Source: PeerJ. 2022 Nov 8;10:e13864. doi: 10.7717/peerj.13864 (PMC9651041; doi:10.7717/peerj.13864)
Supplement: Supplemental Information 1 [file peerj-10-13864-s001.docx]

Supplementary Table 1: Search strategies

| Database | Search Strategy |
| --- | --- |
| PubMed | **#1**- (((((((Tooth Erosion[MeSH Terms]) OR Tooth Erosion[Title/Abstract]) OR Erosion, Tooth[Title/Abstract]) OR Erosions, Tooth[Title/Abstract]) OR Tooth Erosions[Title/Abstract]) OR Erosive tooth[Title/Abstract]) OR dental erosion[Title/Abstract]) OR enamel erosion[Title/Abstract]  **#2**-(((Therapy[MeSH Terms]) OR Therapy[Title/Abstract]) OR Treatment[Title/Abstract]) OR disease management[Title/Abstract] OR anti-erosive toothpaste[Title/Abstract]) OR Toothpastes[MeSH Terms]) OR Toothpastes[Title/Abstract]) OR Toothpaste[Title/Abstract]) OR Dentifrices[MeSH Terms]) OR Dentifrices[Title/Abstract]) OR Dentifrice[Title/Abstract]) OR Dental Polishes[Title/Abstract]) OR Polishes, Dental[Title/Abstract]) OR Mouthwashes[MeSH Terms]) OR Mouthwashes[Title/Abstract]) OR Mouth Bath[Title/Abstract]) OR Bath, Mouth[Title/Abstract]) OR Baths, Mouth[Title/Abstract]) OR Mouth Baths[Title/Abstract]) OR Mouth Wash[Title/Abstract]) OR Wash, Mouth[Title/Abstract]) OR Mouth Rinse[Title/Abstract]) OR Mouth Rinses[Title/Abstract]) OR Rinse, Mouth[Title/Abstract]) OR Rinses, Mouth[Title/Abstract]) OR Fluorides, Topical[MeSH Terms]) OR Fluorides, Topical[Title/Abstract]) OR Topical Fluorides[Title/Abstract]) OR Fluoride Varnishes[Title/Abstract]) OR Varnishes, Fluoride[Title/Abstract]  **#3-** ((Saliva, Artificial[MeSH Terms]) OR Saliva, Artificial[Title/Abstract]) OR Artificial Saliva[Title/Abstract]  **#4-** ((((((((((Dental Enamel[MeSH Terms]) OR Dental Enamel[Title/Abstract]) OR Enamel, Dental[Title/Abstract]) OR Enamel[Title/Abstract]) OR Enamels[Title/Abstract]) OR Dental Enamels[Title/Abstract]) OR Enamels, Dental[Title/Abstract]) OR Enamel Cuticle[Title/Abstract]) OR Cuticle, Enamel[Title/Abstract]) OR Cuticles, Enamel[Title/Abstract]) OR Enamel Cuticles[Title/Abstract] |
| Scopus | **#1-** ( TITLE-ABS-KEY ( "Tooth Erosion" ) OR TITLE-ABS-KEY ( "Erosion, Tooth" ) OR TITLE-ABS-KEY ( "Erosions, Tooth" ) OR TITLE-ABS-KEY ( "Tooth Erosions" ) OR TITLE-ABS-KEY ( "Erosive tooth" ) OR TITLE-ABS-KEY ( "dental erosion" ) OR TITLE-ABS-KEY ( "enamel erosion" ) )  **#2-** ( TITLE-ABS-KEY ( therapy ) OR TITLE-ABS-KEY ( treatment ) OR TITLE-ABS-KEY ( "disease management" ) OR TITLE-ABS-KEY ( "anti-erosive toothpaste" ) OR TITLE-ABS-KEY ( toothpastes ) OR TITLE-ABS-KEY ( toothpaste ) OR TITLE-ABS-KEY ( dentifrices ) OR TITLE-ABS-KEY ( dentifrice ) OR TITLE-ABS-KEY ( "Dental Polishes" ) OR TITLE-ABS-KEY ( "Polishes, Dental" ) OR TITLE-ABS-KEY ( mouthwashes ) OR TITLE-ABS-KEY ( "Mouth Bath" ) OR TITLE-ABS-KEY ( "Bath, Mouth" ) OR TITLE-ABS-KEY ( "Baths, Mouth" ) OR TITLE-ABS-KEY ( "Mouth Baths" ) OR TITLE-ABS-KEY ( "Mouth Wash" ) OR TITLE-ABS-KEY ( "Wash, Mouth" ) OR TITLE-ABS-KEY ( "Mouth Rinse" ) OR TITLE-ABS-KEY ( "Mouth Rinses" ) OR TITLE-ABS-KEY ( "Rinse, Mouth" ) OR TITLE-ABS-KEY ( "Rinses, Mouth" ) OR TITLE-ABS-KEY ( "Fluorides, Topical" ) OR TITLE-ABS-KEY ( "Topical Fluorides" ) OR TITLE-ABS-KEY ( "Fluoride Varnishes" ) OR TITLE-ABS-KEY ( "Varnishes, Fluoride" ) )  **#3-** ( TITLE-ABS-KEY ( "Saliva, Artificial" ) OR TITLE-ABS-KEY ( "Artificial Saliva" ) )  **#4-** ( TITLE-ABS-KEY ( "Dental Enamel" ) OR TITLE-ABS-KEY ( "Enamel, Dental" ) OR TITLE-ABS-KEY ( enamel ) OR TITLE-ABS-KEY ( enamels ) OR TITLE-ABS-KEY ( "Dental Enamels" ) OR TITLE-ABS-KEY ( "Enamels, Dental" ) OR TITLE-ABS-KEY ( "Enamel Cuticle" ) OR TITLE-ABS-KEY ( "Cuticle, Enamel" ) OR TITLE-ABS-KEY ( "Cuticles, Enamel" ) OR TITLE-ABS-KEY ( "Enamel Cuticles" ) ) |
| Web of Science | **#1-** TS=("Tooth Erosion") OR ("Erosion, Tooth") OR ("Erosions, Tooth") OR ("Tooth Erosions") OR ("Erosive tooth") OR ("dental erosion") OR ("enamel erosion")  **#2-** TS=(therapy OR treatment OR "disease management" OR "anti-erosive toothpaste” OR toothpastes OR toothpaste OR dentifrices OR dentifrice OR "Dental Polishes" OR "Polishes, Dental" OR mouthwashes OR "Mouth Bath" OR "Bath, Mouth" OR "Baths, Mouth" OR "Mouth Baths" OR "Mouth Wash" OR "Wash, Mouth" OR "Mouth Rinse" OR "Mouth Rinses" OR "Rinse, Mouth" OR "Rinses, Mouth" OR "Fluorides, Topical" OR "Topical Fluorides" OR "Fluoride Varnishes" OR "Varnishes, Fluoride")  **#3-** TS=("Saliva, Artificial" OR "Artificial Saliva")  **#4-** TS=("Dental Enamel" OR "Enamel, Dental" OR enamel OR enamels OR "Dental Enamels" OR "Enamels, Dental" OR "Enamel Cuticle" OR "Cuticle, Enamel" OR "Cuticles, Enamel" OR "Enamel Cuticles") |
| The Cochrane Library | **#1**- ("Tooth Erosion") OR ("Erosion, Tooth") OR ("Erosions, Tooth") OR ("Tooth Erosions") OR ("Erosive tooth") OR ("dental erosion") OR ("enamel erosion")  **#2-** (Therapy) OR (Treatment) OR (“disease management”) OR (“anti-erosive toothpaste”) OR (toothpastes) OR (toothpaste) OR (dentifrices) OR (dentifrice) OR ("Dental Polishes") OR ("Polishes, Dental") OR (mouthwashes) OR ("Mouth Bath") OR ("Bath, Mouth") OR ("Baths, Mouth") OR ("Mouth Baths") OR ("Mouth Wash") OR ("Wash, Mouth") OR ("Mouth Rinse") OR ("Mouth Rinses") OR ("Rinse, Mouth") OR ("Rinses, Mouth") OR ("Fluorides, Topical") OR ("Topical Fluorides") OR ("Fluoride Varnishes") OR ("Varnishes, Fluoride")  **#3-** ("Saliva, Artificial") OR ("Artificial Saliva")  **#4-** ("Dental Enamel") OR ("Enamel, Dental") OR (enamel) OR (enamels) OR ("Dental Enamels") OR ("Enamels, Dental") OR ("Enamel Cuticle") OR ("Cuticle, Enamel") OR ("Cuticles, Enamel") OR ("Enamel Cuticles") |
| LILACS | **#1-** ("Tooth Erosion") OR ("Erosion, Tooth") OR ("Erosions, Tooth") OR ("Tooth Erosions") OR ("Erosive tooth") OR ("dental erosion") OR ("enamel erosion")  **#2-** (Therapy) OR (Treatment) OR (“disease management”) OR (“anti-erosive toothpaste”) OR (toothpastes) OR (toothpaste) OR (dentifrices) OR (dentifrice) OR ("Dental Polishes") OR ("Polishes, Dental") OR (mouthwashes) OR ("Mouth Bath") OR ("Bath, Mouth") OR ("Baths, Mouth") OR ("Mouth Baths") OR ("Mouth Wash") OR ("Wash, Mouth") OR ("Mouth Rinse") OR ("Mouth Rinses") OR ("Rinse, Mouth") OR ("Rinses, Mouth") OR ("Fluorides, Topical") OR ("Topical Fluorides") OR ("Fluoride Varnishes") OR ("Varnishes, Fluoride")  **#3-** ("Saliva, Artificial") OR ("Artificial Saliva")  **#4-** ("Dental Enamel") OR ("Enamel, Dental") OR (enamel) OR (enamels) OR ("Dental Enamels") OR ("Enamels, Dental") OR ("Enamel Cuticle") OR ("Cuticle, Enamel") OR ("Cuticles, Enamel") OR ("Enamel Cuticles") |
| OpenGrey | Tooth erosion AND Therapy AND Artificial saliva |
| Google Scholar | Tooth erosion+Therapy+Artificial saliva-review |
|  |  |
