# Supplementary material for: Treatment for dental erosion: a systematic review of in vitro studies
Source: PeerJ. 2022 Nov 8;10:e13864. doi: 10.7717/peerj.13864 (PMC9651041; doi:10.7717/peerj.13864)
Supplement: Supplemental Information 2 [file peerj-10-13864-s002.docx]

Supplementary Table 2: Discretion for risk assessment of bias according to “The Cochrane Collaboration’s tool for assessing risk of bias (HIGGINS et al., 2011).

| Random sequence generation | |
| --- | --- |
| Criteria for judgment of  "Low risk" of bias. | Researchers describe the randomization process for patient selection at the start of the study, such as: |
| Criteria for judgment of  "High risk" of bias. | Researchers describe flaws in the randomization process,. |
| Criteria for judgment of  "Uncertain risk" of bias. | Insufficient information on the sequence generation process to allow judgment of "low risk" or "high risk |
| Allocation concealment | |
| Criteria for judgment of  "Low risk" of bias. | One of the following was used to hide allocation: sealed envelopes and central allocation (including telephone, web or randomized control. |
| Criteria for judgment of  "High risk" of bias. | Allocation based on: a list of random numbers, unsealed or non-sequentially numbered envelopes, date of birth, and any other explicitly unknown procedure. |
| Criteria for judgment of  "Uncertain risk" of bias. | Insufficient information to allow the judgment of 'Low risk' or 'High risk', for example if it is not described in sufficient detail to allow a final judgment. |
| Blinding of participants and personnel | |
| Criteria for judgment of  "Low risk" of bias. | Adequate blinding of results. |
| Criteria for judgment of  "High risk" of bias. | Absence of blinding, incomplete blinding or failures during the process. |
| Criteria for judgment of  "Uncertain risk" of bias. | Insufficient information or lack of information about blinding. |
| EVALUATION OF RESULTS | |
| Criteria for judgment of  "Low risk" of bias. | Blinding of participants and study team |
| Criteria for judgment of  "High risk" of bias. | Absence of blinding, incomplete blinding or failures during the process. |
| Criteria for judgment of  "Uncertain risk" of bias. | Insufficient information or lack of information about blinding. |
| Incomplete outcome data | |
| Criteria for judgment of  "Low risk" of bias. | Any of the following: There is no missing outcome data and there is no lack of balanced outcome data in numbers between intervention groups. |
| Criteria for judgment of  "High risk" of bias. | Any of the following: lack of data on intervention groups and imbalance in numbers between groups of interventions. |
| Criteria for judgment of  "Uncertain risk" of bias. | Any of the following: insufficient reporting to allow "low risk" or "high risk" judgment or when the study does not address this outcome. |
| Selective reporting | |
| Criteria for judgment of  "Low risk" of bias. | Any of the following: The study protocol is available and specified (primary and secondary), or even when not available, it is clear that they include all the expected results. |
| Criteria for judgment of  "High risk" of bias. | Any of the following: Not all pre-specified study results were reported or the study report does not include results for a key outcome that would be expected. |
| Criteria for judgment of  "Uncertain risk" of bias. | Insufficient information to allow judgment of 'Low risk' or 'High risk'. |
| Other bias | |
| Criteria for judgment of  "Low risk" of bias. | The study appears to be free from other sources of bias. |
| Criteria for judgment of  "High risk" of bias. | There is at least one major risk of bias. For example, have a potential source of bias, be fraudulent or any other problem. |
| Criteria for judgment of  "Uncertain risk" of bias. | There may be a risk of bias, but there is insufficient information to assess whether there is a significant risk of bias. |
