# Supplementary material for: Treatment for dental erosion: a systematic review of in vitro studies
Source: PeerJ. 2022 Nov 8;10:e13864. doi: 10.7717/peerj.13864 (PMC9651041; doi:10.7717/peerj.13864)
Supplement: Supplemental Information 4 [file peerj-10-13864-s004.pdf]

Due to the increase in the consumption of acidic beverages, foods, and medicines, there was an increasing increase in dental erosion, which is characterized by the irreversible loss of hard dental tissue, due to dissolution by acids of non-bacterial origin. In this way, effective preventive measures to minimize the effects that these acids have on the teeth, several products, such as toothpaste and mouthwashes, have recently been developed with remineralizing or anti-erosion properties. Today, there are several products on the market for the treatment of dental erosion, most of them composed of fluorides, such as sodium fluoride, stannous fluoride, amine fluoride, among others, each one with its effectiveness.

Due to the wide variety of products that exist today, this systematic review aims to search for studies that make a comparison between the various products and their active compounds, to know which is the most effective to prevent or treat dental erosion. The search for an answer is important because the constant loss of mineralized tissue leaves the tooth more fragile.
